# Integrating Genomic, eQTL, and Mendelian Randomization Analyses to Identify Microglial Drug Targets in Multiple Sclerosis

**DOI:** 10.1111/jcmm.70754

**Published:** 2025-11-14

**Authors:** Wu Yan, Jiang Wen, Wang Jianhong

**Affiliations:** ^1^ Department of Neurology First Affiliated Hospital of Kunming Medical University Kunming P. R. China

**Keywords:** Bayesian colocalization, cis‐eQTL analysis, mendelian randomization (MR), microglia, multiple sclerosis (MS), single‐cell sequencing

## Abstract

Multiple sclerosis (MS) is an autoimmune disease characterised by neuroinflammation and neurodegeneration. This study investigates genetic and immunological factors in MS, focusing on microglial regulation. We analysed differentially expressed genes using RNA sequencing from MS lesions (GSE108000) and plaques (GSE227781), validated with cis‐eQTL analysis, and integrated Mendelian randomisation (MR), SMR, co‐localisation, methylation, and protein–protein interaction (PPI) analyses to assess causal effects on MS risk. We identified five genes—ARHGAP25, HLA‐DRB1, MERTK, MS4A6A, and SYK—linked to MS susceptibility. MR revealed that elevated levels of ARHGAP25 (OR = 1.45), HLA‐DRB1 (OR = 2.24), MERTK (OR = 1.10), MS4A6A (OR = 1.15), and SYK (OR = 1.13) increased MS risk. SMR confirmed a causal link between HLA‐DRB1 and MS, while co‐localisation analysis showed shared variants with MS for HLA‐DRB1 (100%) and SYK (97.93%). Methylation analysis highlighted 10 sites within HLA‐DRB1, and PPI and DrugBank analyses revealed interactions between these genes and multiple proteins or chemicals. This study demonstrates the value of integrating genomic and eQTL data through MR to identify novel MS therapeutic targets, particularly microglial genes, offering potential new avenues for treatment.

## 1 Introduction

MS is a chronic autoimmune disease that leads to inflammation, demyelination, and neurodegeneration within the central nervous system (CNS), progressively impairing neurological function. The disease is characterised by an abnormal immune response, wherein immune cells, including microglia, play a critical role in both driving the pathological processes and potentially mediating repair.

Microglia, the resident immune cells of the CNS, are crucial for homeostasis, injury response, and inflammation regulation. In MS, their activation contributes to disease progression by disrupting the blood–brain barrier, recruiting immune cells, and releasing pro‐inflammatory cytokines, which worsen demyelination and neurodegeneration [1, 2, 3, 4]. However, microglia also promote neuroprotection and remyelination by clearing apoptotic cells and secreting neurotrophic factors [5, 6]. This dual role highlights their central involvement in MS pathology and their potential as therapeutic targets. Modulating microglial activity could provide new strategies for promoting neuroprotection and tissue repair, offering hope for improving MS patient outcomes [2].

Expression Quantitative Trait Loci (eQTL) studies link genetic variants to gene expression, shedding light on disease susceptibility, particularly in MS [7]. Recent research highlights microglia as a key player in MS, with cell‐type‐specific eQTLs in microglia strongly associated with MS risk [8, 9, 10]. eQTL analysis has identified MS‐related genes like *CLECL1* and *CYP24A1*, showing distinct patterns in microglia and emphasising their relevance to the disease [10]. These studies also demonstrate the potential of eQTL data to guide drug target discovery for MS, advancing precision medicine in neuroinflammatory diseases. MR, using genetic variants linked to exposures as IVs, has been applied in diseases like Parkinson's and COVID‐19 [11, 12], but few MR studies have integrated GWAS and eQTL data in MS microglia.

This study aims to explore the molecular mechanisms underpinning microglial involvement in MS, utilising multiple techniques such as RNA sequencing, single‐cell sequencing, and MR to better understand their roles and identify potential therapeutic targets.

## 2 Materials and Methods

### 2.1 Study Design

**FIGURE 1:**
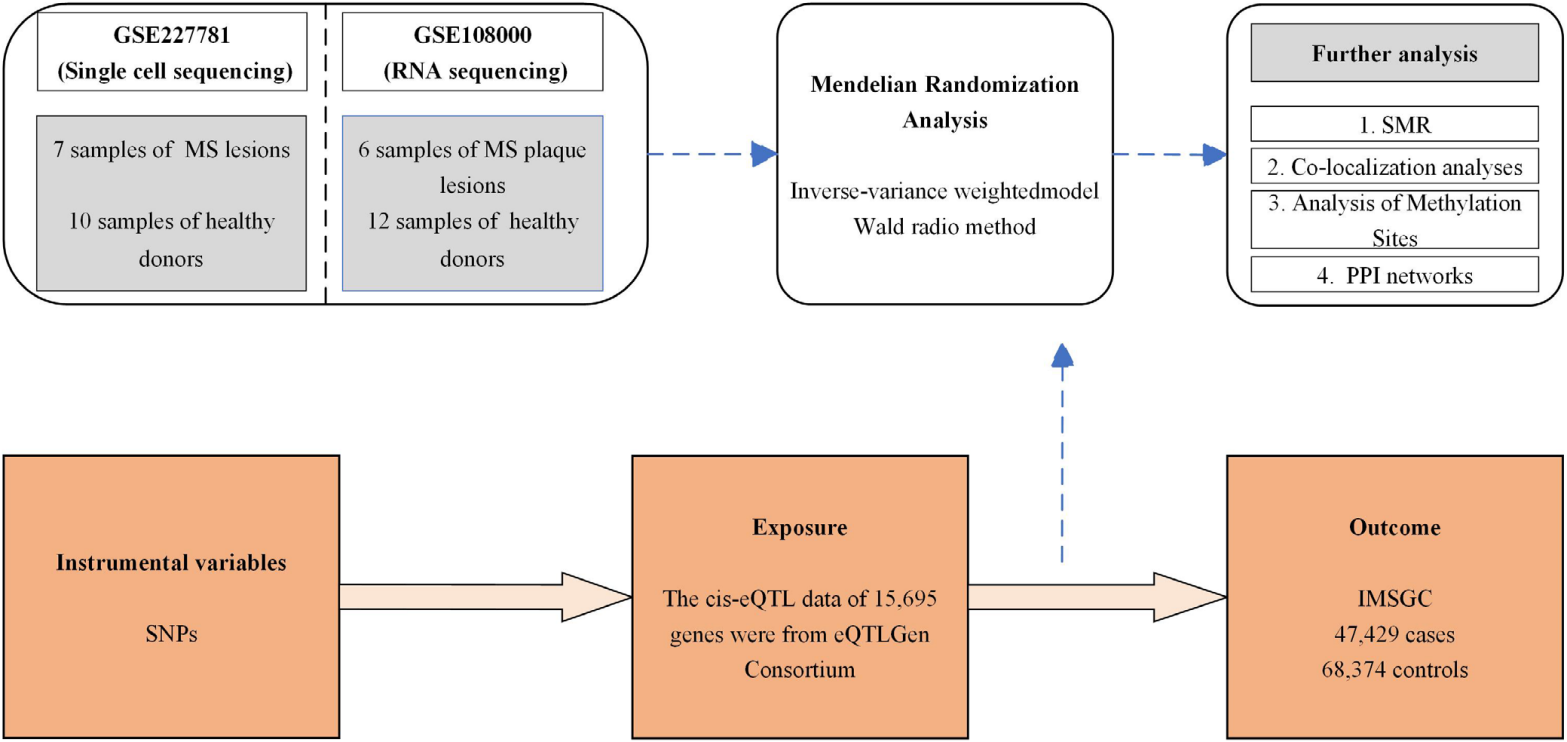
Outlines the study design.

Figure 1 outlines the study design. We analysed marker genes in microglia from MS patients using bulk RNA sequencing data from the GEO database, supplemented with single‐cell sequencing data. To explore the genetic basis of microglial involvement in MS, we utilised cis‐eQTL data for genes, including druggable targets, from the eQTLGen database. MS GWAS data were used as the outcome measure. A two‐sample MR analysis was performed to assess the causal relationship between eQTL gene expression and MS, using carefully selected SNPs as instrumental variables (IVs). Sensitivity analyses were conducted to verify the reliability of our findings. Additionally, we integrated the datasets to identify key genes in MS microglia as potential therapeutic targets, exploring SMR, methylation sites, and interaction networks to validate their relevance to MS pathology.

### 2.2 Public Data Collection and Processing

In this study, we evaluated one bulk RNA sequencing from a chronic active MS lesion of a human from a public dataset (GSE108000 [13]) and one single‐nuclei RNA sequencing (snRNAseq) of human CNS tissue from a public dataset (GSE227781 [14]) from the GEO database (https://www.ncbi.nlm.nih.gov/geo/) with the R package GEOquery (version 4.4.0). Both datasets consisted of RNA sequencing data obtained from brain tissues of MS patients and healthy donors.

The GSE108000 dataset includes gene expression profiles from 7 samples of chronic active MS lesions and 10 samples of white matter from healthy donors. Differentially expressed mRNAs were analysed using the Limma package, with significant genes defined as those with |log2FC| ≥ 1 and *p* < 0.05. The results are summarised in Table S1.

The GSE227781 dataset comprises gene expression profiles from 6 MS plaque lesion samples and 12 white matter samples from 6 healthy donors. After quality control to exclude cells with fewer than 200 genes or with mitochondrial gene content exceeding 10%, the data were normalised, log‐transformed, and scaled to correct for unwanted variations, including cell cycle effects. Dimensionality reduction was performed using principal component analysis (PCA) and t‐distributed stochastic neighbour embedding (t‐SNE). Cell clustering was conducted with the Louvain algorithm, and cell types were annotated based on cluster marker comparisons with published datasets. The single‐cell RNA sequencing (scRNA‐seq) dataset was annotated using the Human Cell Atlas and processed with Seurat (v4.0) in R. Microglia were annotated based on the expression of CSF1R and P2RY12, which are overlapping gene markers identified in CellMarker 2.0, integrating single‐cell sequencing data, experimental studies, and reviews. Differential expression analysis was then performed using the Limma package, applying the same criteria (|log2FC| ≥ 1 and *p* < 0.05), with results presented in Table S2. DEGs were identified using Wilcoxon rank‐sum tests and visualised with t‐SNE plots and volcano plots to highlight cell‐type‐specific expression patterns.

### 2.3 MR Analysis

A two‐sample MR analysis was conducted using eQTL data from the eQTLGen database as the exposure and genome‐wide association study (GWAS) data on MS as the outcome.

The eQTL data, covering 15,695 genes from 31,684 participants, was sourced from the eQTLGen Consortium [15], with 4479 druggable genes identified [16]. Suitable SNPs were selected as IVs with *p*‐values < 5.0 × 10^−8^, and sensitivity analyses were performed.

The primary MS GWAS data from the International Multiple Sclerosis Genetics Consortium (IMSGC), including 115,803 individuals of European ancestry, was used [17]. SNPs with *p*‐values < 1 × 10^−5^ and linkage disequilibrium (LD) *r* ^2^ < 0.001 were included, excluding weak instruments, palindromic alleles, and those with missing outcome data [18, 19].

MR analysis was conducted using the “TwoSampleMR” R package, applying the Wald ratio for single SNP IVs and five methods (IVW, MR‐Egger, weighted median, simple mode, weighted mode) for multiple SNPs. Sensitivity analysis included Cochrane's *Q* test for heterogeneity and MR‐Egger regression for pleiotropy, with *p*‐values < 0.05 indicating significant heterogeneity or pleiotropy.

### 2.4 SMR Analysis

SMR [20] combines GWAS summary statistics and eQTL data to assess pleiotropic relationships between protein expression levels and complex traits [21]. The heterogeneity in dependent instruments (HEIDI) test identifies potential horizontal pleiotropy, with the null hypothesis assuming no pleiotropy, meaning a genetic variant influences multiple traits independently of the investigated trait [22]. Integrating SMR and HEIDI methods helps distinguish whether genetic variants affect phenotypes through protein expression or alternative mechanisms. We obtained the SMR Linux version (1.3.1) from the official website (https://yanglab.westlake.edu.cn/software/smr) [21] on 3 April 2024, using default settings for the analysis.

### 2.5 Bayesian Colocalisation

For eQTL genes that overlapped with DEGs from two RNA sequencing datasets exhibiting significant MR results, colocalization analysis was performed using the “coloc” R package with default prior probabilities. Posterior probabilities assessed five hypotheses, with PPH4 > 0.8 indicating strong colocalization. Variants within ±500 kb of the reference variant were analysed, identifying therapeutic targets for MS.

### 2.6 Analysis of Methylation Sites Associated With Disease by Marker Genes

The study employed a multi‐step bioinformatic and statistical approach to explore the causal relationship between DNA methylation of microglia‐associated genes and MS through MR. CpG sites linked to target genes were identified using Illumina Human Methylation 450 k data, followed by filtering based on gene annotations. Methylation quantitative trait locus (mQTL) data were processed to extract relevant CpG sites, and LD clumping (*r* ^2^ = 0.1, window size = 10,000 kb) was performed to identify independent significant variants using PLINK. Genome‐wide significant variants (*p* < 5e‐8) were retained, and harmonised exposure and outcome datasets were prepared. Instrument strength was assessed using *R* ^2^ and *F*‐statistics (*F* > 10), with results presented as odds ratios and false discovery rate adjustments.

### 2.7 Functional Insights and Network Analyses

To further our understanding, we combined our findings with PPI networks, employing databases such as STRING and DrugBank to investigate potential interactions and their implications for therapy.

## 3 Results

### 3.1 Identification of MS Microglia‐Associated Markers Through RNA Sequencing Analysis

**FIGURE 2:**
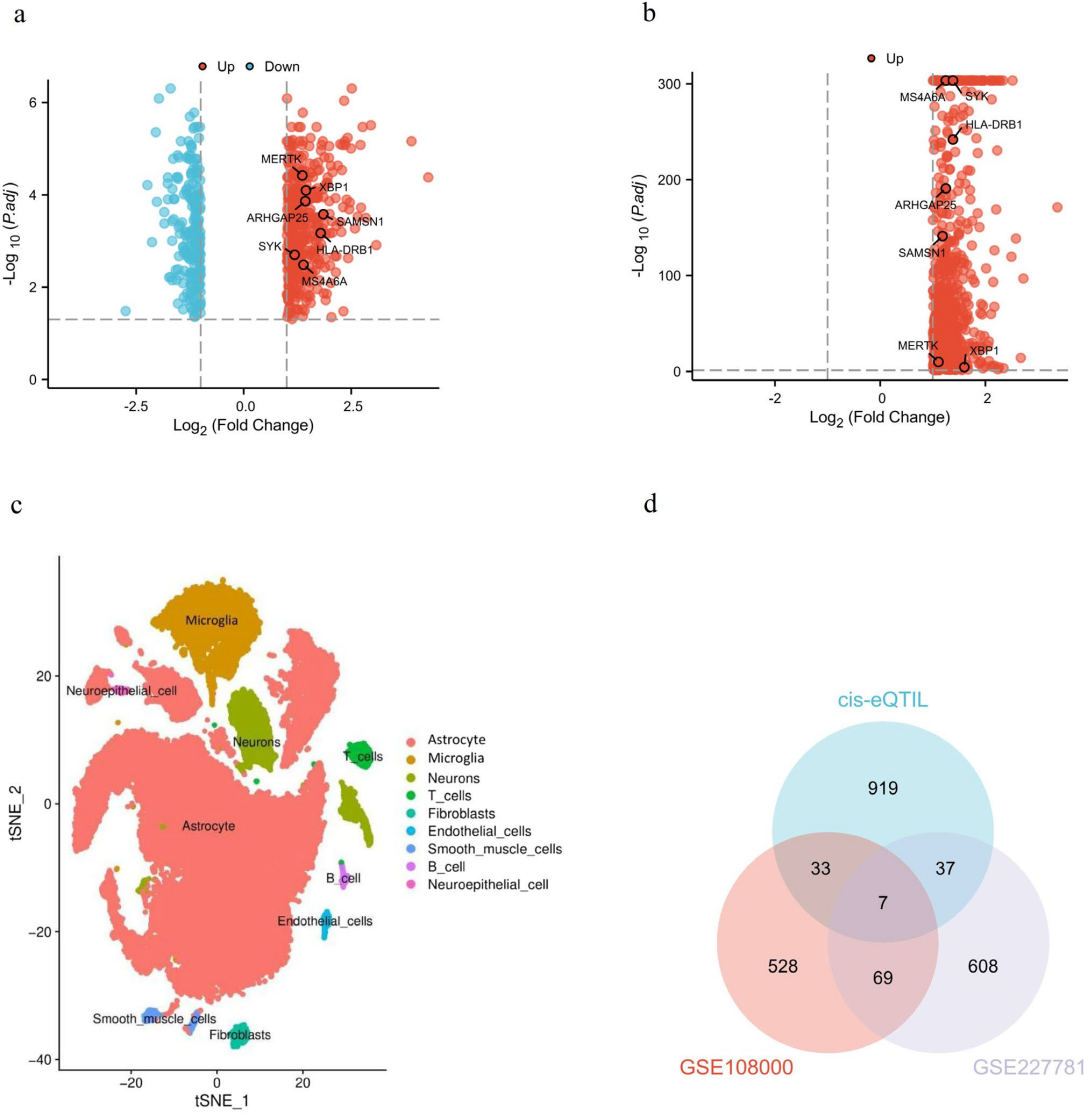
Screening of microglial marker genes in multiple sclerosis (MS). (a, b) Volcano plots showing differentially expressed genes in the GSE108000 (A) and GSE227781. (b) Datasets. (c) Neuronal cell clusters identified through single‐cell RNA sequencing analysis of the GSE227781 dataset. (d) Venn diagram depicting the overlap of marker genes from the GSE108000 and GSE227781 datasets with eQTL‐associated genes.

We employed the limma package to detect mRNAs exhibiting significant differential expression between microglia derived from chronic active MS lesions and white matter from healthy controls of the GSE108000 dataset. Specifically, we identified 428 significantly upregulated mRNAs and 209 significantly downregulated mRNAs (Table S1, Figure 2a) in MS microglia compared to healthy controls.

Our deep dive into scRNA‐seq data of GSE227781 dataset revealed 721 significantly upregulated mRNAs (Table S2, Figure 2b,c) in MS microglia compared to healthy controls.

### 3.2 MR

Based on the established selection criteria for IVs, a total of 15,695 eQTL genes were included in MR analysis (ID refers to Table S3). The *F*‐statistic for all IVs surpassed the threshold of 20, suggesting the absence of weak instrument bias.

Utilising the Inverse Variance Weighting (IVW) approach, our analysis revealed a significant association between the expression levels of 996 genes that are correlated with MS (*p* < 0.05) (Table S4). After intersecting the DEGs identified in GSE108000 and GSE227781, seven genes were found to be commonly associated with MS, namely, *ARHGAP25, HLA‐DRB1, MERTK, MS4A6A, SAMSN1, SYK*, and *XBP1* (Figure 2d). The MR result of the seven genes could be referred to Table 1. Our results demonstrated that elevated levels of *ARHGAP25* (IVW odds ratio [OR] = 1.45, *p* = 0.012), *HLA‐DRB1* (OR = 2.24, *p* = 0.005), *MERTK* (OR = 1.10, *p* < 0.001), *MS4A6A* (OR = 1.15, *p* = 0.027), and *SYK* (OR = 1.13, *p* < 0.001) were associated with an increased risk of MS. Conversely, the odds ratios for *SAMSN1* (OR = 0.74, *p* = 0.020) and *XBP1* (OR = 0.62, *p* < 0.001) were found to be less than 1, suggesting a potential protective role for these factors in MS. This observation contradicts the findings from the GSE108000 and GSE227781 datasets and, therefore, *SAMSN1* and *XBP1* were excluded from further analysis. To be noted, among *ARHGAP25, HLA‐DRB1, MERTK, MS4A6A, and SYK, HLA‐DRB1, MERTK*, and *SYK* are druggable genes [16].

**TABLE 1:** Mendelian randomization analysis of seven candidate genes and their association with multiple sclerosis.

ID	nsnp	Beta	SE	iVW p	OR	OR_lci95	OR_uci95	Heter.Stat	Heter.p	Egger_intercept	Egger_intercept_se	Egger_intercept_pval
ARHGAP25	3	0.37	0.15	0.011	1.45	1.09	1.94	1.69	0.43	0.04	0.04	0.49
HLA‐DRB1	9	0.81	0.28	0.005	2.24	1.28	3.91	892.35	2.53E‐187	0.10	0.18	0.60
MERTK	20	0.10	0.02	4.44E‐05	1.10	1.05	1.16	18.96	0.46	−0.01	0.01	0.53
MS4A6A	13	0.15	0.07	0.03	1.16	1.02	1.32	18.51	0.10	−0.03	0.02	0.21
SAMSN1	4	−0.30	0.13	0.02	0.74	0.57	0.95	3.54	0.32	−0.01	0.03	0.89
SYK	23	0.12	0.03	3.80E‐04	1.13	1.06	1.21	35.10	0.04	−0.01	0.01	0.64
XBP1	8	−0.31	0.09	4.49E‐04	0.73	0.62	0.87	2.78	0.90	−0.01	0.03	0.75

The MR analysis revealed no pleiotropy for the significant genes (*ARHGAP25, HLA‐DRB1, MERTK, MS4A6A, SYK*), supporting the validity of the observed associations with MS risk (Table 2). Cochran's Q test indicated no heterogeneity in the IVs for *ARHGAP25, MERTK*, and *MS4A6A*, suggesting consistent causal effects (Table 2). However, heterogeneity was detected for *HLA‐DRB1* and *SYK*, indicating variability in the genetic effects and warranting further investigation into these associations.

**TABLE 2:** Mendelian randomization results for five candidate genes in multiple sclerosis.

Exposure	Outcome	Method	Number of SNP	*F*‐statistic	Odds ratio (95% CI)	*p*	Heterogeneity	P_h_	Egger intercept	P_*intercept*_
ARHGAP25	MS	MR Egger	3		0.95 (0.41–2.24)	0.93	0.64	0.42	0.041	0.49
		Weighted median	3		1.45 (1.00–2.08)	0.05				
		Inverse variance weighted	3	61.90	1.45 (1.09–1.94)	0.01	1.69	0.43		
HLA‐DRB1	MS	MR Egger	9		1.889 (0.817–4.367)	0.181	854.907	2.615E‐180	0.098	0.597
		Weighted median	9		1.825 (1.696–1.964)	4.778e‐58				
		Inverse variance weighted	9	282.740	2.239 (1.284–3.907)	0.005	892.348	2.525e‐187		
		PRESSO	9		2.239 (1.918–2.561)	0.022				
MERTK	MS	MR Egger	20		1.122 (1.044–1.206)	0.006	18.540	0.421	−0.006	0.533
		Weighted median	20		1.117 (1.055–1.182)	1.264E‐04				
		Inverse variance weighted	20	370.348	1.102 (1.052–1.155)	4.443e‐05	18.956	0.460		
		PRESSO	20		1.102 (1.102–1.092)	0.001				
MS4A6A	MS	MR Egger	13		1.36 (1.04–1.77)	0.05	15.93	0.14	−0.03	0.21
		Weighted median	13		1.11 (0.96–1.28)	0.15				
		Inverse variance weighted	13	167.30	1.16 (1.02–1.32)	0.03	18.51	0.10		
		PRESSO	13		1.16 (1.12–1.19)	0.05				
SYK	MS	MR Egger	23		1.16 (1.03–1.31)	0.03	34.72	0.03	−0.01	0.64
		Weighted median	23		1.09 (1.01–1.18)	0.02				
		Inverse variance weighted	23	253.08	1.13 (1.06–1.21)	3.80E‐04	35.10	0.04		
		PRESSO	23		1.13 (1.12–1.14)	1.78E‐03				

### 3.3 SMR Analysis and Colocalisation Analysis

The *p*‐value obtained from the SMR analysis was below 0.05 for both whole blood and brain tissue, indicating a causal relationship between HLA‐DRB1‐encoded proteins and MS. To assess the presence of pleiotropy, we performed the HEIDI test. Our results revealed that the *p*‐value for the HEIDI test (p_HEIDI) associated with *HLA‐DRB1* in whole blood was greater than 0.05, suggesting no pleiotropy for SNPs linked to HLA‐DRB1‐encoded proteins in this tissue. However, p_HEIDI in brain tissue was less than 0.05, indicating the presence of pleiotropy in this context (refer to Table 3).

**TABLE 3:** SMR analysis of five candidate genes in multiple sclerosis.

ProbeChr	Gene	Probe_bp	TopSNP	TopSNP_chr	TopSNP_bp	A1	A2	Freq	b_GWAS	SE_GWAS	p_GWAS	b_eQTL	SE_eQTL	p_eQTL	b_SMR	SE_SMR	p_SMR	p_HEIDI	nsnp_HEIDI
2	ARHGAP25	68,906,733	rs7605681	2	69,051,320	A	G	0.50	−0.02	0.03	4.59e‐01	−0.08	0.01	7.41e‐09	0.28	0.37	4.62e‐01	4.54e‐01	3
2	MERTK	112,656,056	rs10203902	2	112,780,825	A	G	0.08	0.002	0.05	9.72e‐01	0.35	0.04	3.54e‐18	0.01	0.15	9.72e‐01	5.65e‐01	14
6	HLA‐DRB1	32,546,546	rs113568276	6	32,513,127	A	G	0.26	−0.31	0.04	3.74e‐17	−0.24	0.02	2.24e‐35	1.31	0.19	3.20e‐12	1.93e‐01	20
11	MS4A6A	59,939,081	rs636147	11	59,957,092	T	C	0.56	0.01	0.03	7.86e‐01	0.13	0.01	6.40e‐20	0.06	0.24	7.86e‐01	8.79e‐01	20

In our investigation of genes exhibiting noteworthy MR outcomes, we performed a colocalisation analysis to assess the likelihood that the causal variant responsible for the cis‐eQTL is also implicated in MS outcomes. The findings from this colocalisation assessment suggested a probable shared causal variant between MS susceptibility and two prominent genes, namely *HLA‐DRB1* and *SYK*, with posterior probabilities exceeding 0.80% (*HLA‐DRB1*: 100.00%, *SYK*: 97.93%) (refer to Table 4). Therefore, *HLA‐DRB1* and *SYK* have been identified as potential pharmacological targets for modulating the risk of MS, with *HLA‐DRB1*, in particular, being highlighted as a key target based on findings from both SMR and colocalisation analyses.

**TABLE 4:** Bayesian co‐localization analysis for five candidate genes in multiple sclerosis.

ID	nsnps	PP.H0.abf	PP.H1.abf	PP.H2.abf	PP.H3.abf	PP.H4.abf
HLA‐DRB1	11	0	5.71e‐116	0	0	1
SYK	24	0	0.02	0	5.57e‐05	0.98

### 3.4 Analysis of Methylation Sites Associated With Disease by Marker Genes

Analysis of methylation sites in five microglia‐associated genes identified 10 methylation sites within the *HLA‐DRB1* gene, all of which are located on chromosome 6, encompassing 207 SNPs associated with MS (Table S5). Specifically, we analysed the methylation status of these 10 CpG sites, with five located in the CpG island, three in the shore, and two in the shelf regions. Methylation of CpG sites within the CpG island is typically linked to gene silencing and may contribute to the downregulation of *HLA‐DRB1* expression, potentially altering immune responses [23]. Methylation in the shore and shelf regions could influence enhancer activity or chromatin remodelling, modulating gene expression in a more subtle or context‐dependent manner [23]. These findings suggest that methylation of *HLA‐DRB1* plays a significant role in the regulation of microglia immune functions, with altered methylation patterns in these regions potentially contributing to the pathogenesis of MS.

### 3.5 PPI Network

**FIGURE 3:**
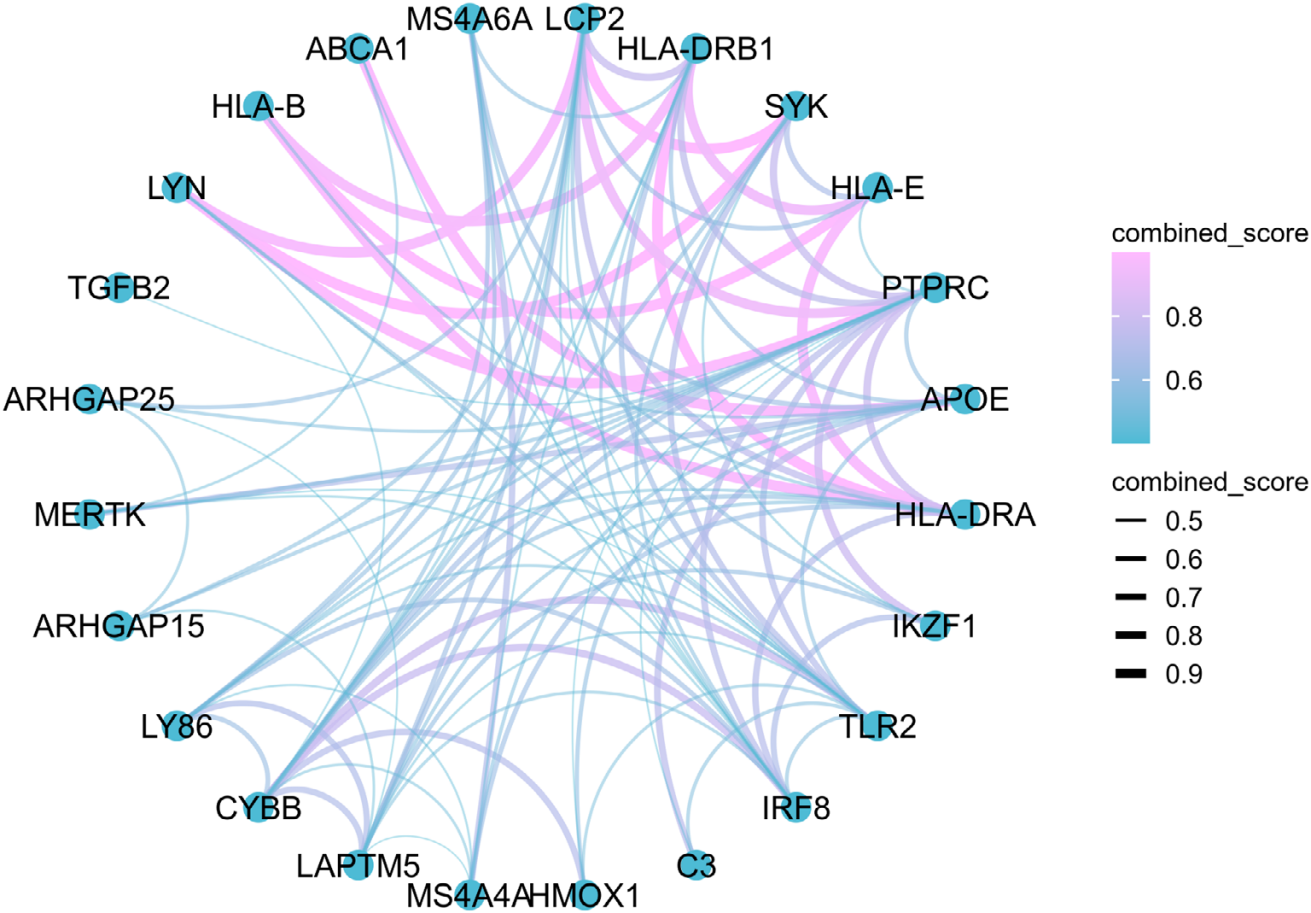
Protein–Protein Interaction (PPI) Network Analysis. Nodes represent the target genes, with the colour and thickness of the connecting lines indicating the strength of the correlation between nodes. The colour gradient ranges from blue (low correlation) to red (high correlation), and the line thickness increases with the degree of correlation.

We conducted a comprehensive PPI analysis of five microglia‐associated target genes—*ARHGAP25*, *HLA‐DRB1*, *MERTK*, *MS4A6A*, and *SYK*—using the STRING database. To enhance the analysis, overlapping genes identified from the GSE108000 and GSE227781 datasets were incorporated into the PPI network, highlighting the involvement of these five targets. Gene selection was based on the presence of direct or indirect interactions with other nodes in the PPI network, as shown in Figure 3 and Table S6. The PPI analysis revealed frequent interactions among the five genes, with HLA‐DRB1, MS4A6A, and SYK interacting with more than five other proteins.

To investigate potential pharmacological interventions targeting these proteins, we queried the DrugBank database. Notably, ARHGAP25, HLA‐DRB1, and MS4A6A were not associated with any approved drugs (see Table S7). In contrast, MERTK was associated with four therapeutic agents, including Fostamatinib, Denfivontinib, and Tamnorzatinib, while SYK was linked to 15 drugs, such as Tamatinib, Staurosporine, Mivavotinib, Lanraplenib, Gusacitinib, Fostamatinib, Entospletinib, Ellagic acid, Cevidoplenib, Cerdulatinib, and others (see Table S7 for the full list). These findings indicate that existing therapeutic agents targeting MERTK and SYK may influence MS pathophysiology, highlighting the need for further investigation into their potential applications in MS.

## 4 Discussion

This study utilises RNA sequencing of microglia in MS, combined with single‐cell sequencing data, two‐sample MR, and Bayesian colocalisation techniques to identify potential therapeutic proteins and biomarkers. The candidate genes—*ARHGAP25*, *HLA‐DRB1*, *MERTK*, *MS4A6A*, and *SYK*—are crucial in MS pharmacodynamics and therapeutic strategies. Our findings suggest that microglial genetic regulation plays a critical role in MS susceptibility, supporting microglial pathway targeting as a therapeutic approach. This aligns with [8], who identified microglia‐specific cis‐eQTLs at MS risk loci, and [10], who highlighted variants like CLECL1 and CYP24A1. The integration of eQTLGen and GTEx data, as shown by [9], emphasises the role of tissue‐ and cell‐type‐specific variants in disease risk. Our results, consistent with [24], highlight the complex regulation of microglial genes and their causal relationship with MS susceptibility. These findings suggest that modulating microglial gene expression could mitigate neuroinflammation and MS progression, warranting further exploration to refine therapeutic strategies for MS and other neurodegenerative disorders.

Our integrative analysis highlights *HLA‐DRB1* as a key genetic and immunological factor in MS pathogenesis. *HLA‐DRB1* is highly expressed in activated microglia, playing a critical role in antigen presentation and inflammatory modulation [25]. Consistent with previous studies, *HLA‐DRB1* expression in microglia correlates with disease activity and neurological impairment, supporting its involvement in MS progression [26]. The *HLA‐DRB1* locus, particularly the *HLA‐DRB115:01* allele, is strongly associated with increased MS susceptibility, especially in females [25, 27]. The genetic complexity of *HLA‐DRB1*, including interactions between risk alleles (e.g., *HLA‐DRB115:01*) and protective alleles (e.g., HLA‐DRB1*01), underscores its role in MS susceptibility and immune regulation [27, 28]. Our analysis shows *HLA‐DRB1* upregulation in MS lesion microglia, with colocalization analyses identifying shared causal variants in CNS tissues. Additionally, *HLA‐DRB1*'s effect on MS can be modulated by its methylation sites, suggesting potential therapeutic strategies targeting *HLA‐DRB1* pathways.

MERTK (Mer receptor tyrosine kinase) plays a critical role in microglial function and MS pathogenesis. It mediates efferocytosis, essential for resolving inflammation and tissue repair [29]. In microglia, MERTK signalling regulates the balance between pro‐inflammatory and anti‐inflammatory responses, a key mechanism in MS and other neurodegenerative diseases [30]. Activation of MERTK by ligands like Gas6 and Protein S triggers signalling pathways that reduce pro‐inflammatory cytokines, such as TNF‐α and IL‐6, contributing to demyelination [30, 31]. Recent studies show that *HLA‐DRB1* and *MERTK* genotypes independently influence MS risk by modulating monocyte behaviour, with the DR15 haplotype linked to reduced proportions of CD14+ MERTK+ monocytes, and the *MERTK* SNP rs7422195 affecting MERTK expression in monocytes [32]. Our analysis identifies *MERTK* as a key gene in MS risk, suggesting its dysregulation contributes to neuroinflammation in MS lesions. Additionally, PPI and DrugBank analyses highlight *MERTK*'s interactions with several proteins and chemicals, supporting its potential as a therapeutic target for reducing inflammation and protecting against neuronal damage.

ARHGAP25, a Rho GTPase‐activating protein, plays a significant role in MS progression by regulating cellular motility, phagocytosis, and cytokine production through its modulation of Rac1 and related pathways [33]. Our findings show ARHGAP25's involvement in microglial activation, with its expression in MS lesions linked to inflammatory pathways like I‐κB/NF‐κB signalling, which regulates IL‐1β production [34]. Additionally, ARHGAP25 participates in non‐immune processes, such as glycolysis regulation via AKT/mTOR signalling and osteoblast differentiation [35, 36]. MR analysis revealed a causal link between *ARHGAP25* expression and MS risk, while PPI networks highlighted its key role in microglial functions. These results align with its protective roles in other inflammatory and neurodegenerative conditions, where ARHGAP25 suppresses excessive cellular migration and pro‐inflammatory cytokines [37]. These findings suggest *ARHGAP25* as a promising therapeutic target to modulate microglial activity and inflammation in MS, warranting further investigation into its molecular interactions and potential druggability for personalised MS therapies.

MS4A6A, a key regulator of microglial activation and immune response, plays a role in neurodegenerative diseases like Alzheimer's and glioblastoma [38, 39]. Our study identifies MS4A6A as a major inflammation modulator in MS, where its elevated expression in microglia contributes to chronic inflammation and disease progression [38]. Similar findings in glioblastoma suggest it promotes microglial activation and tumour aggressiveness [39]. MS4A6A regulates soluble TREM2 (sTREM2), influencing neuroinflammation in Alzheimer's [40], and genetic variations in its locus exacerbate inflammation in neurodegenerative diseases [41]. Missense variants in MS4A6A also impact sTREM2 levels, linking it to neuroinflammation and highlighting its therapeutic potential [42]. Thus, MS4A6A is a critical target for therapeutic strategies in MS and other neurodegenerative diseases.

The limitation of this study is the reliance on publicly available datasets, such as eQTLGen and GEO databases, which may not capture the full genetic diversity of MS patients across different populations. Additionally, while our MR and colocalisation analyses suggest causality, functional validation in experimental models is required to confirm the roles of the identified genes in MS pathogenesis. Further studies incorporating larger, diverse cohorts and in vivo models are needed to validate and expand upon these findings.

## 5 Conclusion

This study highlights the significant role of microglial genes in MS susceptibility and pathogenesis, emphasising the potential of targeting microglial pathways for therapeutic intervention. Our findings provide a foundation for the development of novel strategies aimed at modulating microglial activity, with implications for improving MS treatment. Future research should focus on further elucidating the functional roles of the identified genes and exploring potential therapeutic approaches to mitigate neuroinflammation and enhance neuroprotection in MS.

## Author Contributions

**Jiang Wen:** conceptualization (lead), data curation (equal), formal analysis (equal), investigation (equal), methodology (equal), project administration (equal), resources (equal), software (equal), supervision (lead), validation (equal), visualization (equal), writing – original draft (equal), writing – review and editing (equal). **Wang Jianhong:** data curation (equal), formal analysis (equal), methodology (equal), project administration (equal), resources (equal), software (equal), validation (equal), visualization (equal), writing – original draft (equal), writing – review and editing (equal). **Wu Yan:** conceptualization (lead), data curation (equal), formal analysis (equal), funding acquisition (lead), investigation (equal), methodology (equal), project administration (lead), resources (equal), software (equal), supervision (lead), validation (equal), visualization (equal), writing – original draft (equal), writing – review and editing (equal).

## Disclosure

*Declarations*: The authors assert that the study was carried out without any commercial or financial affiliations that could be interpreted as a possible conflict of interest.

*Ethics Approval and Consent to Participate*: This study did not require additional ethical approval or informed consent, as it solely utilised publicly available summary data. The original GWAS, from which these data were derived, had obtained the necessary ethical approval and participant consent.

## Consent

The authors have nothing to report.

## Conflicts of Interest

The authors declare no conflicts of interest.

## Supporting information


**Table S1.** jcmm70754‐sup‐0001‐TableS1.xlsx.

**Table S2.** jcmm70754‐sup‐0002‐TableS2.xlsx.

**Table S3.** jcmm70754‐sup‐0003‐TableS3.xlsx.

**Table S4.** jcmm70754‐sup‐0004‐TableS4.xlsx.

**Table S5.** jcmm70754‐sup‐0005‐TableS5.xlsx.

**Table S6.** jcmm70754‐sup‐0006‐TableS6.xlsx.

**Table S7.** jcmm70754‐sup‐0007‐TableS7.xlsx.

## Data Availability

All data supporting the findings of this study are publicly available from the following repositories: RNA‐seq data: Gene Expression Omnibus (GEO) under accession numbers GSE108000 (MS lesions) and GSE227781 (MS plaques). GWAS summary statistics: International Multiple Sclerosis Genetics Consortium (IMSGC) and eQTLGen Consortium (eQTLGen database).
